# Punicalin Modulates Angiogenesis and Tumor Microenvironment-Related Processes in Triple-Negative Breast Cancer and Endothelial Cells

**DOI:** 10.3390/ijms27031533

**Published:** 2026-02-04

**Authors:** Maria Carmen Banqueri-Pegalajar, Joel D. Posligua-García, Carlos Ulises Cárdenas-Vela, Manuel Bernal, Miguel Ángel Medina

**Affiliations:** 1 Department of Molecular Biology and Biochemistry, Faculty of Science, Universidad de Málaga, Andalucía Tech, 29071 Málaga, Spain; mcbp@uma.es (M.C.B.-P.); joel.posgar@uma.es (J.D.P.-G.); ulisescardenas@uma.es (C.U.C.-V.); 2 Molecular Bases of Biological Systems (SIBIUMA) Group, Instituto de Investigación Biomédica de Málaga y Plataforma en Nanomedicina-IBIMA Plataforma Bionand, 29590 Málaga, Spain; 3 Center for Biomedical Network Research in Rare Diseases (CIBERER), Instituto de Salud Carlos III, 28029 Madrid, Spain

**Keywords:** punicalin, triple-negative breast cancer, tumor microenvironment, angiogenesis, cell migration, oxidative stress, autophagy, natural bioactive compounds, high content screening

## Abstract

The tumor microenvironment plays a critical role in cancer progression, with oxidative stress, autophagy, angiogenesis, and cell migration acting as tightly interconnected processes. Natural bioactive compounds have emerged as promising modulators of these pathways; however, their cell type-specific effects within the TME remain poorly understood. In this study, we investigate the effects of punicalin on triple-negative breast cancer and endothelial cells, with a focus on redox homeostasis and autophagy as upstream regulatory mechanisms. Punicalin reduced oxidative stress in MDA-MB-231 cells under basal conditions and strongly attenuated hydrogen peroxide-induced stress, whereas HMEC-1 cells exhibited concentration- and condition-dependent reactive oxygen species (ROS) modulation. Autophagy assays revealed no significant modulation in tumor cells, while a consistent and pronounced decrease in autophagic activity was observed in endothelial cells under both basal and nutrient-deprivation conditions. Functionally, punicalin decreased tumor cell migration and impaired HMEC-1 migration, while HUVEC migration remained largely unaffected. Tube formation assays demonstrated significant inhibition of angiogenic capacity. Taken together, these findings demonstrate that punicalin selectively modulates oxidative stress and autophagy, leading to functional alterations in migration and angiogenesis. By highlighting its selective impact on microvascular endothelial cells while sparing normal endothelium, this study provides a strong rationale for further preclinical evaluation of punicalin.

## 1. Introduction

Breast cancer is the most diagnosed malignancy among women worldwide and remains a leading cause of cancer-related morbidity and mortality. Among its subtypes, triple-negative breast cancer (TNBC) is a particularly aggressive form, defined by the lack of expression of estrogen receptors (ERs), progesterone receptors (PRs), and human epidermal growth factor receptor 2 (HER2). TNBC accounts for approximately 10–20% of all breast cancer cases and is more prevalent in younger women [1]. The absence of these receptors prevents the use of endocrine and HER2-targeted therapies, leaving chemotherapy and, in selected cases, immunotherapy as the main treatment options. Despite these approaches, clinical outcomes for TNBC remain poor compared with other breast cancer subtypes [2]. Consequently, there is a critical need to identify novel compounds or therapeutic strategies that can improve disease management and outcomes for patients with this aggressive malignancy [3].

Despite early conceptual recognition of the importance of the tumor microenvironment (TME), notably through the “seed and soil” hypothesis, mainstream cancer research throughout much of the mid-20th century remained largely centered on tumor cells and their genetic alterations as the primary determinants of cancer development [4]. A paradigm shift emerged in the 1990s, driven by advances in molecular biology and signal transduction, which revealed that microenvironmental components actively regulate gene expression in both tumor and stromal cells. These findings demonstrated that the TME is not a passive bystander but a dynamic contributor to tumor growth and progression [5]. This evolving perspective led to formal acknowledgment by major cancer research institutions in the early 2000s, including the National Cancer Institute, which identified the TME as a priority area of investigation [6].

Studying the TME is critically important because cancer development, progression, and therapeutic response are not determined by tumor cells alone, but by their dynamic interactions with surrounding non-malignant components. TME consists of stromal cells, immune cells, blood vessels, extracellular matrix, and soluble factors, all of which actively shape tumor behavior [7]. TNBC is highly heterogeneous at molecular and cellular levels, and this heterogeneity is amplified by interactions with the TME. Stromal cells, immune cells, and extracellular matrix components actively shape tumor cell plasticity, invasiveness, and metastatic potential. Because TNBC lacks estrogen, progesterone, and HER2 receptors, these microenvironment-driven signaling pathways often become dominant regulators of disease progression [8].

A major source of TME heterogeneity lies in tumor-associated vascular cells. Quiescent endothelial cells are induced to generate new blood vessels derived from existent vessels in a process called angiogenesis. Although angiogenesis is a physiological process, cancer cells activate the so-called “angiogenic switch”. Notch, Neuropilin, Robo, Eph-A/B, VEGF, angiopoietin and FGF pathways are activated by tumor cells facilitating malignant cells access to nutrients and oxygen and its spread throughout the body using vasculature [9,10]. Thus, metastasis—the biological process by which cancer cells spread from a primary tumor to distant, non-adjacent organs or tissues, where they establish secondary tumors—is highly related to angiogenesis [11,12,13]. Both hallmarks of cancer involve proliferation, migration and differentiation/dedifferentiation processes [14]. These shared features enable the search of compounds that might modulate both processes in hopes of treating TNBC.

Autophagy stress and oxidative stress are cellular processes that highly influence TME state and cancer prognosis. Autophagy plays a dual role in tumor progression as either a prooncogenic or an antioncogenic process, depending on the context. Autophagy limits proliferation and might induce anoikis in metastatic cells but also promotes metabolic reprogramming, immune evasion and secondary tumor growth [15]. These roles establish a clear strategy of inhibiting autophagy of tumor cells to limit tumor growth and prevent new metastases in late stages. Tumor-associated endothelial cells upregulate autophagy resisting hypoxia and promoting angiogenesis. Deficiencies in endothelial autophagy leads to the typical aberrant endothelial phenotype associated with the TME. These differential effects should also be considered when designing a therapeutic strategy [16].

Oxidative stress also plays a dual role in tumor progression [11,13,17]. An increase in reactive oxygen species (ROS) promotes transformation, genomic instability, proliferation and metastasis in malignant cells, but a too high increase in ROS can also induce cell death via apoptosis and ferroptosis [18,19]. ROS are produced both extra- and intracellularly in TME, shaping communication between malignant and stromal cells. Tumor-associated endothelial cells have a basal level of ROS that promote angiogenesis by positive regulation of HIF1α and VEGF. This highlights why usually antioxidant compounds tend to be cancer-preventing and are studied as plausible drugs for therapeutic intervention [11,19,20].

When researching for novel drugs, natural compounds present some advantages over synthetic compounds. Natural compounds tend to affect several targets and to exhibit a reduced profile of adverse effects. Thus, the search for natural compounds that modulate TME-related processes emerges as a promising strategy towards pharmacological research [21,22]. Polyphenols are a group of natural molecules present in plant-derived foods and drinks which usually exhibit antioxidant activity and the ability to modulate metabolic pathways and cellular signaling which contribute to their protective and therapeutic effects [23].

**Figure 1:**
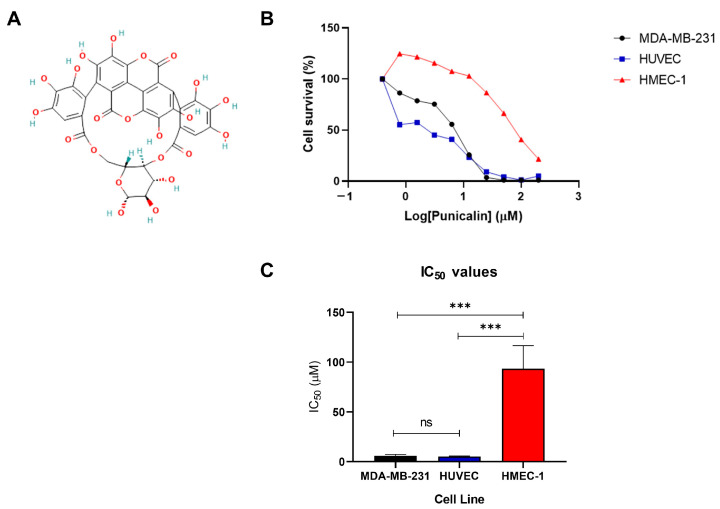
Punicalin differentially reduces cell viability in tumor and endothelial cell lines. (**A**) Chemical structure of punicalin. Chemical structure of punicalin obtained from the PubChem database (PubChem CID: 5464368). (**B**) Dose–response curves showing cell viability after 72 h of treatment with increasing concentrations of punicalin in MDA-MB-231 (triple-negative breast cancer), HMEC-1 (microvascular endothelial), and HUVECs (primary endothelial), as determined by the MTT assay. (**C**) Estimated IC_50_ values of punicalin for each cell line. Data represent three independent biological experiments, each performed in quadruplicate, and are expressed as mean ± standard deviation (SD). Statistical significance was assessed using two-way ANOVA, with significance levels indicated as *** *p* < 0.001. The corresponding survival curves including variability across independent experiments (mean ± SD) are shown in Supplementary Figure S3.

Punicalin (Figure 1A) (molecular formula C_34_H_22_O_22_; molecular weight 782.5 g/mol) is a natural compound corresponding to a type of polyphenols called ellagitanins. It is found in pomegranates and other ellagitannin-containing fruits and plants that exerts bioactivities such as antioxidant, anti-inflammatory and antitumoral properties [24]. However, despite these reported bioactivities, the effects of punicalin on autophagy regulation and angiogenesis within the tumor microenvironment have not yet been investigated. In particular, to the best of our knowledge, no previous studies have examined the impact of punicalin on autophagy and oxidative stress in tumor-associated endothelial cells, nor addressed its potential cell type-specific effects using comparative tumor–endothelial models.

In this study, we investigated how punicalin modulates tumor microenvironment-related processes, oxidative stress, autophagy, and cell migration, in MDA-MB-231 cells, a model of triple-negative breast cancer, and in HMEC-1 cells, a model for tumor-associated angiogenesis. Given the established roles of oxidative stress and autophagy in tumor progression and angiogenic regulation, we hypothesized that punicalin may act as a modulator of tumor-associated cellular processes by targeting these pathways, thereby potentially limiting tumor progression and angiogenesis.

To evaluate this hypothesis, we first performed a live-cell high-content screening (HCS) using an Operetta system to evaluate whether punicalin modulates autophagy and oxidative stress in tumor and endothelial cells. Cell viability was assessed by MTT assays to define biologically relevant concentration ranges. Based on the HCS findings, the functional consequences of punicalin treatment were subsequently investigated using wound healing assays to assess cell migration and Matrigel-based tube formation assays to evaluate its impact on angiogenic capacity.

## 2. Results

### 2.1. In Vitro Cytotoxic Effects of Punicalin Reveal Differential Sensitivity Between Tumor and Endothelial Cells

Treatment with punicalin for 72 h resulted in a concentration-dependent reduction in cell viability in all three cell lines analyzed (MDA-MB-231, HMEC-1, and HUVECs), as determined by the MTT assay (Figure 1B). However, marked differences in sensitivity to punicalin were observed between tumor and endothelial cells.

MDA-MB-231 cells exhibited high sensitivity to punicalin, with a steep dose–response curve and an estimated IC_50_ of 5 µM, indicating potent cytotoxic activity in these TNBC cells. Similarly, HUVECs showed a comparable sensitivity profile, with an IC_50_ value of 5 µM, reflecting a strong response to punicalin in these primary mesovascular endothelial cells. In contrast, the microvascular endothelial cell line HMEC-1 displayed substantially lower sensitivity to punicalin treatment. The dose–response curve for HMEC-1 was shifted to the right, and the estimated IC_50_ was 100 µM, suggesting a markedly higher tolerance to the compound compared with both MDA-MB-231 and HUVECs (Figure 1C).

These results demonstrate a cell type-dependent cytotoxic effect of punicalin, with pronounced activity in TNBC cells and primary mesovascular endothelial cells, while transformed microvascular endothelial cells show relative resistance. This differential response suggests that punicalin selectively affects an endothelial phenotype more closely associated with angiogenic activation, while transformed microvascular endothelial cells, which better reflect the adaptive characteristics of tumor-associated endothelium, show reduced sensitivity.

### 2.2. Punicalin Selectively Inhibits Autophagy in Endothelial Cells Under Basal and Starvation Conditions

Given that several bioactive compounds previously described as modulators of autophagy have also demonstrated antitumoral and antiangiogenic properties, we next investigated whether punicalin could influence autophagic activity in tumor and endothelial cells. Autophagy was analyzed using a HCS approach, which allows dynamic, multiparametric, and single-cell resolution assessment of intracellular processes over time.

Based on the differential sensitivity observed in the cytotoxicity assays, subsequent mechanistic studies were performed in MDA-MB-231 cells and HMEC-1 endothelial cells. HMEC-1 were selected as a more representative in vitro model of microvascular endothelium within the TME, which plays a central role in angiogenesis, and because they exhibited a broader resistance window to punicalin compared to primary endothelial cells (HUVECs), enabling the evaluation of sub-cytotoxic effects.

**Figure 2:**
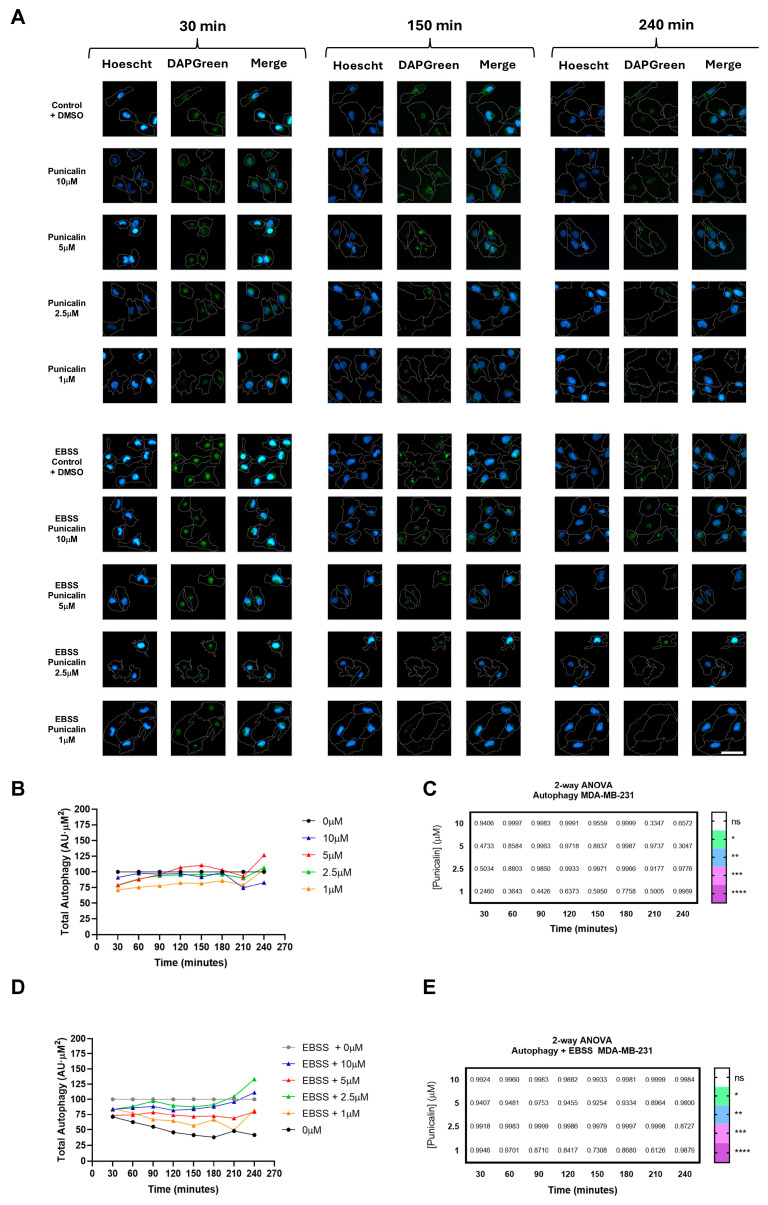
High-content analysis of autophagy in MDA-MB-231 cells treated with punicalin. (**A**) Representative HCS images showing MDA-MB-231 cells stained with Hoechst 33342, CellMask Deep Red, and DAPGreen under basal and starvation conditions. (**B**) Quantification of basal autophagic activity over time at different concentrations of punicalin. (**C**) Heatmap representation of statistical significance for basal autophagy. (**D**) Quantification of starvation-induced autophagy over time. (**E**) Heatmap representation of statistical significance for starvation-induced autophagy. Data represent mean of three independent experiments. Statistical significance is indicated (* *p* < 0.05, ** *p* < 0.01, *** *p* < 0.001; **** *p* < 0.0001 and ns, not significant). A version of (**B**,**D**) quantifications including mean ± SD is depicted in Supplementary Figure S4C,D. Scale bars: 50 µm.

Autophagic activity was evaluated in MDA-MB-231 and HMEC-1 cells under basal and nutrient deprivation conditions using EBSS. In MDA-MB-231 cells, treatment with punicalin did not significantly alter autophagy at any tested concentration, either under basal or starvation conditions, as illustrated in the representative HCS images (Figure 2A) and quantified in basal (Figure 2B,C) and induced conditions (Figure 2D,E).

**Figure 3:**
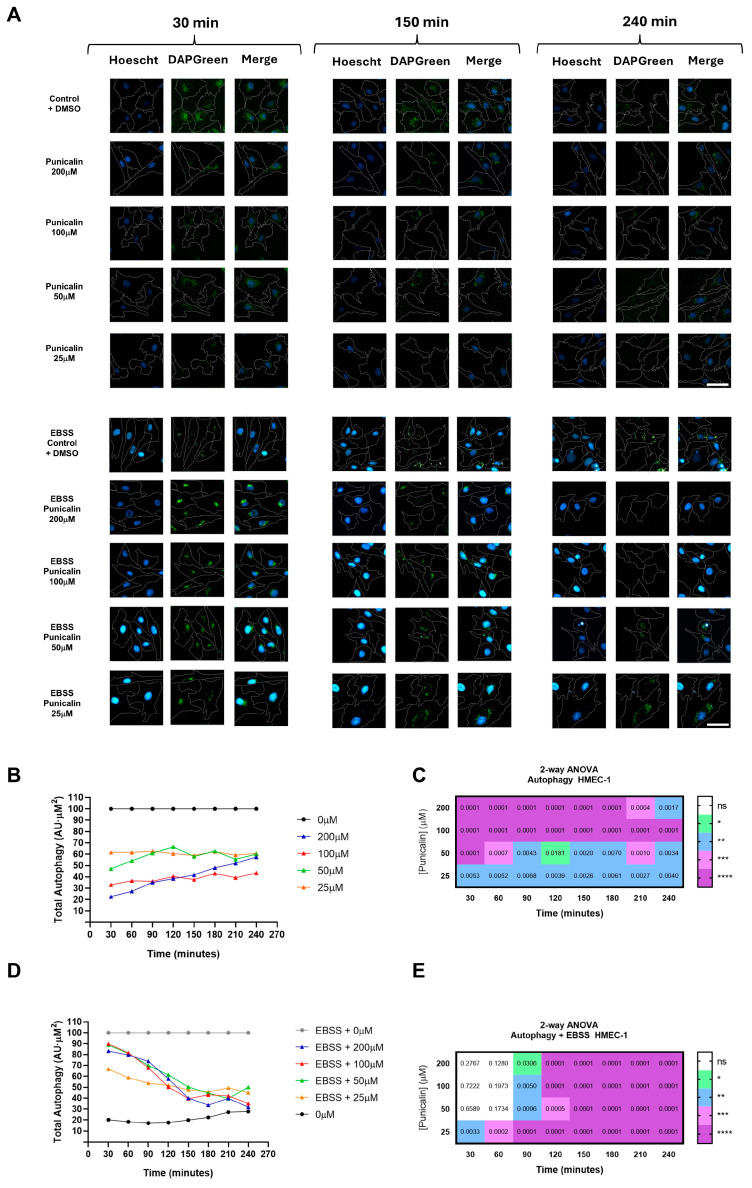
High-content analysis of autophagy in HMEC-1 cells treated with punicalin. (**A**) Representative HCS images showing HMEC-1 cells stained with Hoechst 33342, CellMask Deep Red, and DAPGreen under basal and starvation conditions. (**B**) Quantification of basal autophagic activity over time at different concentrations of punicalin. (**C**) Heatmap representation of statistical significance for basal autophagy. (**D**) Quantification of starvation-induced autophagy over time. (**E**) Heatmap representation of statistical significance for starvation-induced autophagy. Data represent mean of three independent experiments. Statistical significance is indicated (* *p* < 0.05, ** *p* < 0.01, *** *p* < 0.001; **** *p* < 0.0001 and ns, not significant). A version of (**B**,**D**) quantifications including mean ± SD is depicted in Supplementary Figure S4A,B. Scale bars: 50 µm.

In contrast, HMEC-1 cells exhibited a marked decrease in autophagic activity at all tested concentrations under both basal and starvation conditions (Figure 3A). Under basal conditions, autophagy was reduced up to ~80% at the highest concentration (Figure 3B,C), while lower concentrations caused ~40% inhibition. Starvation-induced autophagy was also significantly attenuated, with final time-point levels restored to basal control values (Figure 3D,E). Heatmaps indicate statistically significant differences over time.

These results reveal a cell type-specific and dynamic effect of punicalin, decreasing autophagy by punicalin in endothelial cells, while tumor cells remained largely unaffected. The temporal profiles further suggest that punicalin modulates autophagic activity in a concentration- and time-dependent manner.

### 2.3. Punicalin Modulates Oxidative Stress in Tumor and Endothelial Cells

Since autophagy and oxidative stress are tightly interconnected processes in both cancer cells and the tumor-associated endothelium, we next evaluated the impact of punicalin on intracellular ROS levels using the HCS-based approach. The effect of punicalin on intracellular oxidative stress was evaluated in MDA-MB-231 and HMEC-1 cells under basal and H_2_O_2_-induced conditions.

**Figure 4:**
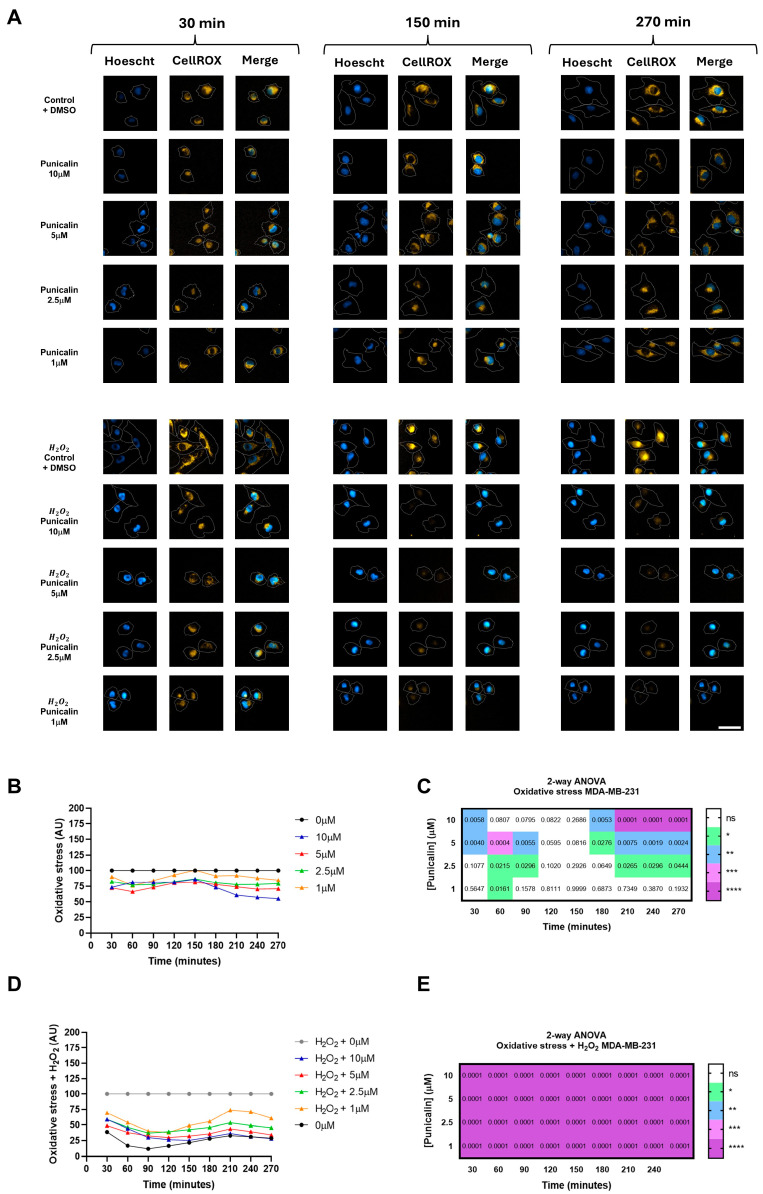
High-content analysis of oxidative stress in MDA-MB-231 cells treated with punicalin. (**A**) Representative HCS images showing MDA-MB-231 cells stained with Hoechst 33342, CellMask Deep Red, and CellROX Deep Orange under basal and H_2_O_2_-induced conditions. (**B**) Quantification of basal oxidative stress over time at different concentrations of punicalin. (**C**) Heatmap of statistical significance for basal oxidative stress. (**D**) Quantification of H_2_O_2_-induced oxidative stress over time. (**E**) Heatmap of statistical significance for H_2_O_2_-induced oxidative stress. Data represent mean of three independent experiments. Statistical significance is indicated (* *p* < 0.05, ** *p* < 0.01, *** *p* < 0.001; **** *p* < 0.0001 and ns, not significant). The corresponding mean ± SD values across independent experiments are shown in Supplementary Figure S5C,D. Scale bars: 50 µm.

In MDA-MB-231 cells, treatment with punicalin progressively reduced oxidative stress over the course of the experiment (Figure 4A).

Under basal conditions, punicalin treatment led to a progressive reduction in intracellular ROS levels in MDA-MB-231 cells, reaching decreases of up to 50% at the final time points compared to untreated controls (Figure 4B,C). Under H_2_O_2_-induced oxidative stress, punicalin significantly attenuated ROS accumulation, reducing intracellular ROS by up to 60% and effectively restoring levels to those observed under basal control conditions (Figure 4D,E). These effects were time-dependent and consistently observed across the tested concentration range, indicating a robust antioxidant response in tumor cells.

**Figure 5:**
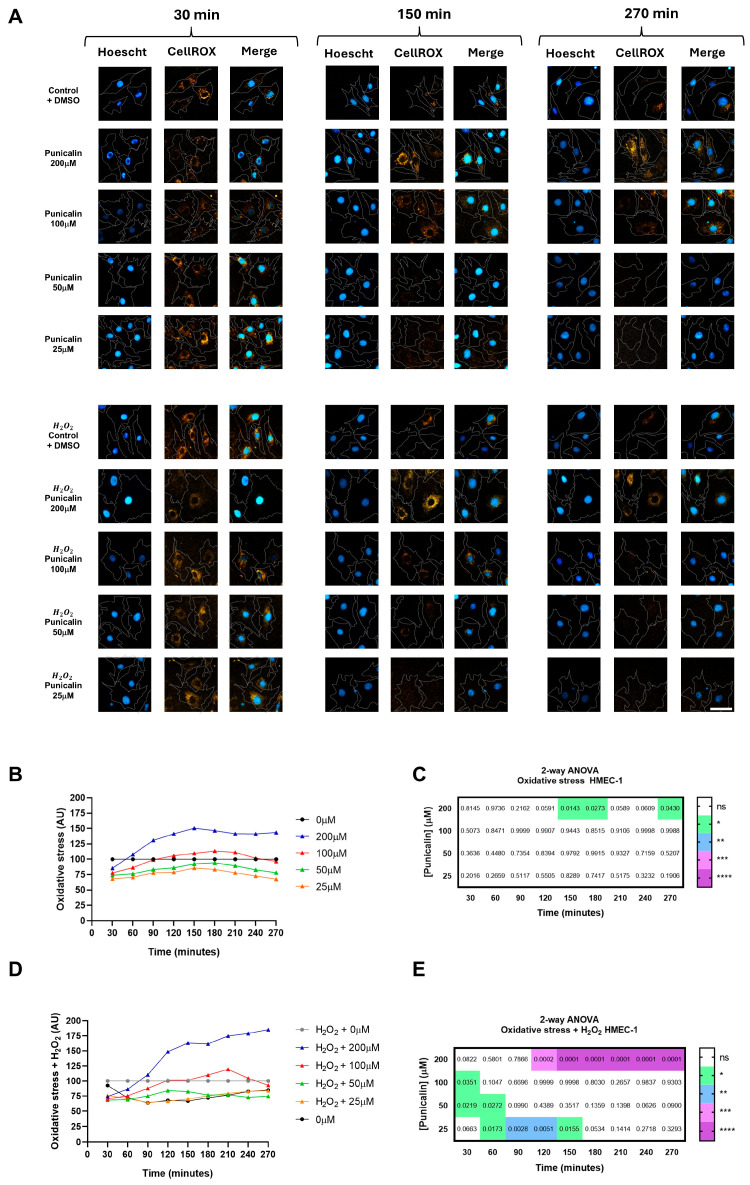
High-content analysis of oxidative stress in HMEC-1 cells treated with punicalin. (**A**) Representative HCS images showing HMEC-1 cells stained with Hoechst 33342, CellMask Deep Red, and CellROX Deep Orange under basal and H_2_O_2_-induced conditions. (**B**) Quantification of basal oxidative stress over time at different concentrations of punicalin. (**C**) Heatmap of statistical significance for basal oxidative stress. (**D**) Quantification of H_2_O_2_-induced oxidative stress over time. (**E**) Heatmap of statistical significance for H_2_O_2_-induced oxidative stress. Data represent mean of three independent experiments. Statistical significance is indicated (* *p* < 0.05, ** *p* < 0.01, *** *p* < 0.001; **** *p* < 0.0001 and ns, not significant). The corresponding mean ± SD values across independent experiments are shown in Supplementary Figure S5A,B. Scale bars: 50 µm.

In contrast, HMEC-1 cells displayed a markedly different, concentration- and condition-dependent response to punicalin (Figure 5A). Under basal conditions, only the highest concentration induced a significant increase in ROS over time, reaching approximately 50% above control levels. A similar pro-oxidant effect was observed under H_2_O_2_-induced stress, where ROS levels increased by up to 100% relative to induced controls (Figure 5B,C). Notably, lower concentrations transiently reduced ROS at specific time points under induced conditions, indicating partial and temporally restricted antioxidant activity (Figure 5D,E).

These findings demonstrate a clear cell type-specific modulation of oxidative stress by punicalin, characterized by a predominantly antioxidant effect in TNBC cells, but a dual, concentration-dependent behavior in endothelial cells, where low concentrations exert transient antioxidant effects while higher concentrations promote ROS accumulation.

### 2.4. Punicalin Partially Inhibits Tumor and Endothelial Cell Migration

The effects of punicalin on cell migration were evaluated in MDA-MB-231, HMEC-1, and HUVECs using a wound-healing assay.

**Figure 6:**
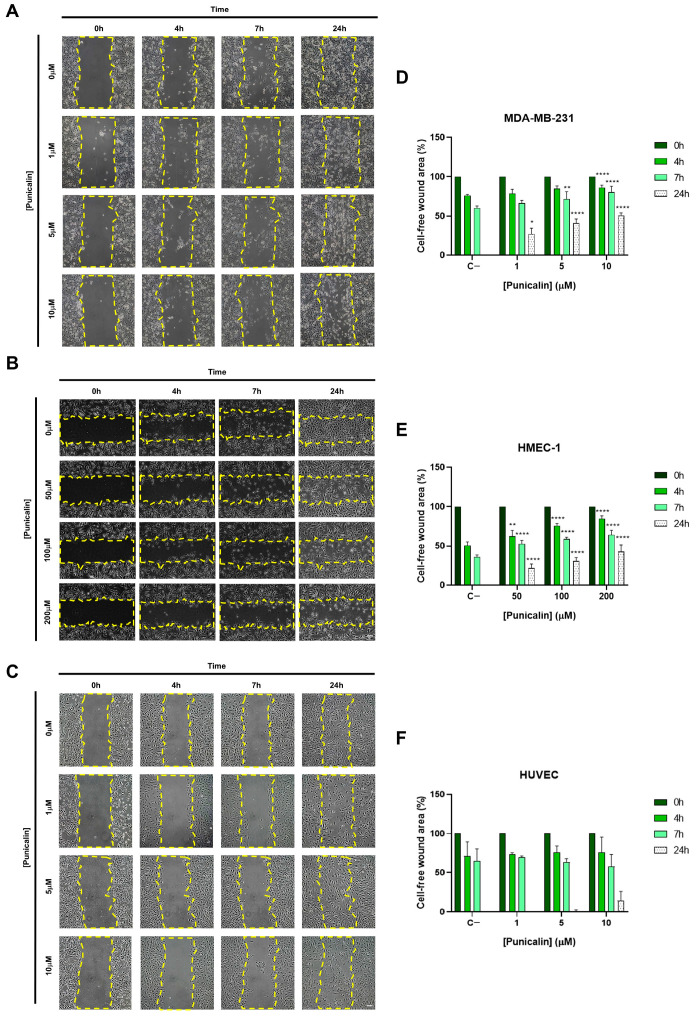
Effects of punicalin on cell migration in MDA-MB-231, HMEC-1, and HUVECs. (**A**) Representative images of MDA-MB-231 cells at indicated time points and concentrations. (**B**) Representative images of HMEC-1 cells. (**C**) Representative images of HUVECs. (**D**) Quantification of MDA-MB-231 migration over time. (**E**) Quantification of HMEC-1 migration over time. (**F**) Quantification of HUVEC migration over time. Dotted yellow lines in (**A**–**C**) represent the limit of “wounds” a t time zero for each condition. Data represent mean ± SD of three independent experiments. Statistical significance was determined for each time point and concentration compared to untreated controls (* *p* < 0.05; ** *p* < 0.01 and **** *p* < 0.0001). Scale bars: 100 µm.

In TNBC MDA-MB-231 cells, migration was partially inhibited in a concentration- and time-dependent manner. The highest concentration caused significant inhibition at all time points, while intermediate and low concentrations showed significant effects only at later times (7–24 h), indicating a gradual reduction in tumor cell motility (Figure 6A,B).

In HMEC-1 cells, punicalin induced a consistent and significant partial inhibition of migration at all concentrations and time points, demonstrating a robust effect on microvascular endothelial cell motility (Figure 6C,D).

In contrast, mesovascular HUVECs were largely unaffected, with no significant changes observed at any concentration or time (Figure 6E,F).

These results highlight a selective inhibitory effect on tumor-associated microvascular endothelial cells (HMEC-1) while sparing normal mesovascular endothelial cells (HUVECs), consistent with the cell type-specific effects observed in autophagy and oxidative stress assays. This selectivity suggests that punicalin could exert antiangiogenic effects in the TME without compromising normal endothelium.

### 2.5. Punicalin Inhibits Endothelial Tube Formation In Vitro

Given the selective inhibition of endothelial cell migration observed in HMEC-1 cells, we next assessed whether punicalin could affect the final step of the angiogenic process. The endothelial tube formation in Matrigel allows the evaluation of the ability of endothelial cells to organize themselves into capillary-like structures, providing a functional readout of potential antiangiogenic activity.

**Figure 7:**
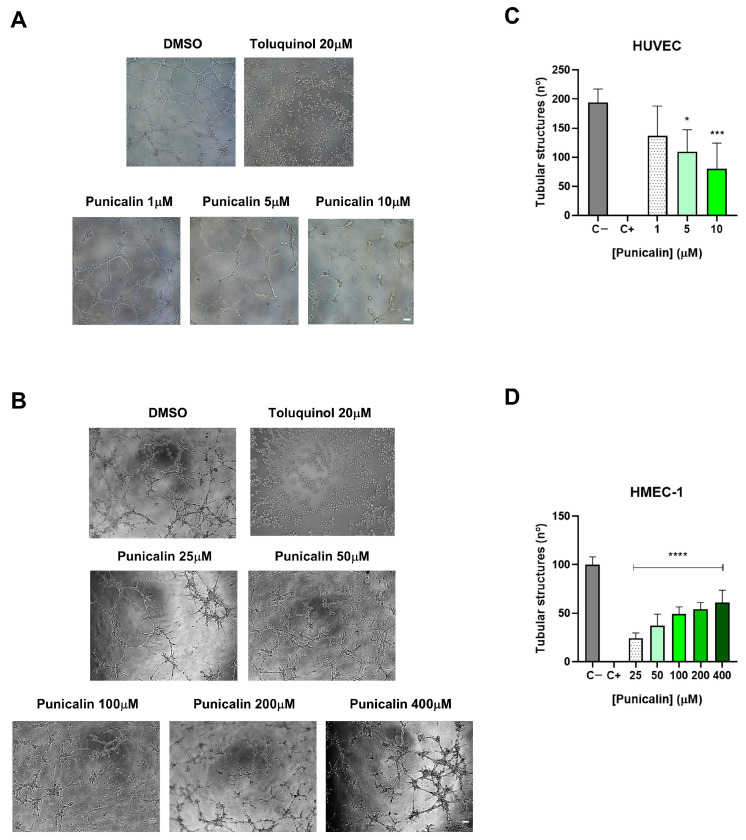
Effects of punicalin on in vitro endothelial tube formation. (**A**) Representative images of HUVEC tube formation at different concentrations of punicalin. (**B**) Quantification of HUVEC tube formation. (**C**) Representative images of HMEC-1 tube formation. (**D**) Quantification of HMEC-1 tube formation. Data represent mean ± SD of at least three independent experiments. Statistical significance was determined compared to untreated controls (* *p* < 0.05; *** *p* < 0.001, and **** *p* < 0.0001). Scale bars: 100 µm.

In HUVECs, punicalin significantly inhibited tube formation at 5 µM and 10 µM, as shown by the quantification of network structures (Figure 7A,B).

In HMEC-1, a more pronounced inhibitory effect was observed, with significant reductions in tube formation at all concentrations tested. Interestingly, the strongest inhibition was detected at the lowest concentration, suggesting a non-linear dose–response relationship in these tumor-associated microvascular endothelial cells (Figure 7C,D).

These results confirm that punicalin exerts functional antiangiogenic effects, selectively impairing endothelial network formation, with a more marked effect in tumor-associated HMEC-1 compared to primary cells (HUVECs). The data are consistent with the previous observations on cell migration, autophagy, and oxidative stress, supporting the potential of punicalin as a selective inhibitor of tumor-associated angiogenic processes.

## 3. Discussion

The present study provides a comparative analysis of the effects of punicalin on breast cancer cells and endothelial cells, revealing distinct cell type-specific responses across multiple functional endpoints. By integrating viability, autophagy, oxidative stress, migration, and angiogenesis assays, our results highlight differential modulation of stress-adaptive pathways in tumor versus endothelial compartments. These findings indicate that punicalin induces cell type-specific cytotoxic effects, differentially impacting tumor and endothelial cells, and underscore the need to consider both cell types when evaluating compounds targeting the tumor microenvironment.

Although punicalin has been primarily characterized as an ellagitannin with antioxidant and anti-inflammatory properties, emerging evidence indicates that it also exerts biological activity in cancer-related contexts. Previous studies have reported that punicalin can suppress proliferation, migration, and invasion of breast cancer cells, and promote apoptotic responses, with redox modulation proposed as a contributing mechanism [25,26]. However, the dynamic regulation of oxidative stress and autophagy by punicalin, as well as its impact on endothelial cell function and angiogenesis within the TME remained poorly defined.

### 3.1. Punicalin and Autophagy

Autophagy is a process that recycles intracellular components to maintain metabolic homeostasis and cellular integrity under stress conditions such as nutrient deprivation, hypoxia, or oxidative pressure. Its functional role in cancer is highly context dependent and can act both as a tumor suppressive mechanism during early carcinogenesis and as a pro survival adaptation in established tumors, enabling cancer cells to endure metabolic and therapeutic stress [27,28]. Beyond tumor cells, autophagy also plays a critical role within the TME, where it regulates the behavior of stromal and endothelial cells, influencing angiogenesis, vascular function, and immune interactions [10,29].

In TNBC cells, high-content screening analysis revealed no significant changes in autophagy levels under either basal conditions or following nutrient deprivation induced by EBSS, despite the pronounced cytotoxic effect of punicalin observed in viability assays (IC_50_ ≈ 5 µM). This lack of detectable modulation may reflect the elevated basal autophagic flux characteristic of TNBC cells, which supports metabolic flexibility and survival in nutrient-limited and hostile microenvironments [28]. Autophagy-related proteins (ARPs) have been shown to exert multifaceted roles in TNBC, functioning not only as mediators of metabolic adaptation but also as contributors to therapeutic resistance and disease progression, highlighting the plasticity and context dependency of autophagic regulation in this tumor subtype [28,30].

The absence of inducible changes in autophagy in MDA-MB-231 cells under our experimental conditions may indicate that basal autophagy is already sufficient to meet metabolic demands, or that alternative survival pathways dominate over nutrient-induced autophagic responses. Indeed, previous studies have demonstrated that TNBC cells often rely on constitutive autophagy as a stress-adaptive mechanism and that effective therapeutic strategies frequently require combinatorial approaches targeting autophagy alongside other signaling pathways, such as PI3K/AKT/mTOR, to overcome resistance and induce cell [27,30]. These observations are consistent with our findings, suggesting that the cytotoxic effects of punicalin in TNBC cells may be mediated predominantly through autophagy-independent mechanisms.

In line with this interpretation, previous reports have suggested that the anticancer effects of punicalin in breast cancer models are associated mainly with apoptosis induction and modulation of survival signaling pathways rather than direct regulation of autophagic flux. In particular, punicalin has been shown to affect ROS levels and apoptosis-related markers in TNBC cells, supporting the notion that its cytotoxic activity may be driven by redox-sensitive and non-autophagic mechanisms [26].

In contrast, endothelial cells exhibited a markedly different response. In HMEC-1 cells, punicalin treatment led to a significant reduction in autophagy levels under both basal and nutrient-deprived conditions, with the effect being more pronounced at later time points and under EBSS-induced stress, coinciding with the emergence of a rounded/detaching morphology compatible with severe stress. Endothelial cell-intrinsic autophagy is known to be essential for maintaining metabolic flexibility, redox homeostasis, and adaptive responses to hypoxia and nutrient scarcity within the TME. These functions are essential for endothelial cell survival and angiogenic capacity in vivo, and their dysregulation can compromise vascular function [10,29,31].

The observed decrease in autophagy suggests that punicalin may impair stress-adaptive autophagy in endothelial cells, thereby limiting their ability to cope with nutrient deprivation and promoting cytotoxicity. This interpretation aligns with evidence indicating that modulation of endothelial autophagy profoundly influences angiogenic phenotypes, vascular remodeling, and endothelial cell survival within tumors [10,29]. Moreover, recent work has identified autophagy in tumor endothelial cells as a key regulator of vascular–immune crosstalk, acting as a functional checkpoint that shapes vessel structure and immune cell infiltration [31].

Reduced autophagy in endothelial cells may have direct implications for angiogenic functions such as migration, tube formation, and network stability. Autophagy supports endothelial responses to metabolic stress and facilitates the dynamic remodeling required for angiogenesis, in part by sustaining ATP production and redox balance during vessel sprouting and growth [10]. In line with this concept, our high-content screening analyses revealed that, at later time points, the combination of nutrient deprivation and punicalin treatment was associated with a marked increase in the rounding of endothelial cells that could indicate cell death and loss of analyzable cells, indicative of an impaired stress-adaptive capacity. Failure to activate these adaptive pathways can render endothelial cells more vulnerable to stress and less capable of executing angiogenic programs, consistent with the partial inhibition of tube formation and migration observed in our functional assays.

In tumor cells, the apparent autonomy from inducible autophagic modulation despite active basal autophagy may represent an adaptive survival strategy, whereby autophagic flux is already optimized to withstand environmental stress. This could contribute to resistance against perturbations targeting the autophagy machinery and may explain why functional endpoints, such as migration, did not correlate directly with autophagy modulation in MDA-MB-231 cells. Autophagy is tightly interconnected with key signaling networks, including mTOR, AMPK, and redox-regulatory pathways, and its modulation can influence not only cell survival but also communication between tumor cells and stromal components within the TME [27].

Our set of results on autophagy suggest that cell type specific modulation of autophagy—with endothelial cells showing impaired adaptive autophagy while tumor cells maintain basal flux—may contribute to differential susceptibility and functional outcomes relevant to angiogenesis and TME adaptation. These findings underscore the importance of considering distinct autophagic states and regulatory mechanisms across cell populations within the tumor ecosystem when interpreting pharmacological effects and therapeutic potential [14,28,29].

### 3.2. Punicalin, Oxidative Stress and Redox Regulation

Punicalin has been reported to exhibit context-dependent redox activity, acting predominantly as an antioxidant in several biological systems, while under certain conditions promoting ROS accumulation associated with cytotoxic responses in cancer cells. Studies using punicalin or punicalin-based formulations have shown modulation of intracellular ROS levels alongside activation of apoptotic signaling pathways in breast cancer models, supporting a close link between its biological activity and redox regulation [32].

Oxidative stress is a central regulator of breast cancer progression, influencing not only tumor cell behavior but also stromal and endothelial functions within the TME. Recent evidence highlights the dual role of reactive oxygen species (ROS) in breast cancer, whereby moderate ROS levels promote tumor growth and angiogenesis, while excessive oxidative stress can impair cell viability and vascular function [33,34]. Importantly, tumor-associated endothelial cells have been shown to exhibit elevated oxidative stress signatures together with enhanced antioxidant defense mechanisms, reflecting an adaptive redox balance that supports endothelial survival and angiogenic activity within breast tumors [11,35]. Moreover, the ROS ATM CHK2 axis has been shown to stabilize HIF-1α under hypoxic conditions, promoting angiogenesis, which aligns with the observed sensitivity of tumor-associated endothelial cells to redox changes [36].

Oxidative stress has been identified as a key driver of the angiogenic switch within the TME, acting as a signaling cue that promotes endothelial activation and neovascularization [37]. Beyond their intracellular effects, ROS have been increasingly recognized as key regulators of microenvironmental signaling. Recent reviews emphasize that redox signaling actively shapes intercellular communication within the TME, influencing endothelial remodeling and angiogenic responses through stress-adaptive pathways [17,38,39]. In this context, ROS function as signaling mediators of tumor–endothelial crosstalk rather than mere byproducts of tumor metabolism, reinforcing their relevance as therapeutic targets within the TME.

Consistent with these observations, our results demonstrate that punicalin differentially modulates intracellular ROS levels in tumor and endothelial cells. In TNBC MDA-MB-231 cells, punicalin reduced ROS levels at later time points under both basal and H_2_O_2_-induced conditions, indicating a sustained antioxidant effect in tumor cells. In contrast, in HMEC-1 cells, ROS modulation was concentration- and condition-dependent: lower concentrations transiently decreased oxidative stress, whereas the highest concentration induced a progressive increase in ROS levels, particularly under oxidative stress conditions. This behavior closely parallels the adaptive redox regulation described for tumor-associated endothelial cells and supports the concept that finely tuned ROS levels act as critical mediators of angiogenic signaling and migratory functions [11,33,34,35,38].

Disruption of this finely balanced redox state has been associated with impaired endothelial adaptation and defective angiogenic responses, supporting the concept that endothelial cells are especially sensitive to perturbations in ROS-mediated signalling [39]. Such sensitivity of endothelial cells to redox imbalance is consistent with previous reports identifying oxidative stress as a key determinant of angiogenic competence [40]. The concentration-dependent modulation of ROS in HMEC-1 cells may account for the lack of a dose-dependent inhibition of tube formation. At lower concentrations, punicalin exerts antioxidant effects that more effectively suppress angiogenic activity, whereas higher concentrations induce a pro-oxidant state that attenuates this inhibitory effect. This suggests that the angiogenic switch in these microvascular endothelial cells may be more sensitive to reductions in ROS levels than to their upregulation. This interpretation aligns with earlier studies demonstrating that moderate ROS levels act as pro-angiogenic signals, whereas angiogenesis is inhibited primarily when oxidative stress exceeds a critical threshold [37,41]. Higher untested punicalin concentrations might therefore lead to stronger inhibition of tube formation by further increasing ROS beyond the tolerable threshold [34].

Interestingly, recent mechanistic studies have suggested that punicalin can interact with redox-regulatory proteins such as protein disulfide isomerase A3 (PDIA3), indicating that it may influence endothelial redox homeostasis through protein-folding and oxidative stress-related pathways [42]. Although these mechanisms have not been explored in endothelial cells, they provide a plausible molecular basis for the differential redox sensitivity observed in HMEC-1 cells in response to punicalin treatment.

From a therapeutic standpoint, these findings are highly relevant. The antioxidant effect observed in tumor cells may limit ROS-driven signaling pathways involved in tumor progression, while the selective disruption of redox homeostasis in tumor-associated endothelial cells may compromise angiogenic capacity. As proposed previously [35], targeting the delicate balance between pro-oxidant and antioxidant mechanisms within the TME represents a promising antitumor strategy. Rather than globally suppressing oxidative stress, selectively interfering with redox-regulated communication within the TME may provide therapeutic benefit [35,38]. In this context, our data suggest that punicalin exploits differential ROS sensitivity between tumor cells and tumor-associated endothelium, selectively impairing endothelial functions critical for angiogenesis while sparing normal endothelial cells, consistent with the concept of ROS as finely tuned signaling mediators in cancer [33,34,38].

### 3.3. Therapeutic Perspectives and Future Directions

The differential effects of punicalin on oxidative stress and autophagy in tumor versus endothelial cells observed in our study highlight the importance of considering both tumor-intrinsic and microenvironmental responses when evaluating potential anticancer compounds. By demonstrating that punicalin selectively impairs adaptive autophagy and modulates redox homeostasis in endothelial cells, while maintaining basal autophagic flux in TNBC cells, our work provides a mechanistic framework for understanding its cell type-specific effects and supports the rationale for targeting tumor vasculature alongside cancer cells.

Several aspects must be considered when evaluating the translational potential of punicalin. It is worth noting that punicalin has a relatively high molecular weight (782.5 g/mol), exceeding the optimum range typically associated with favorable bioavailability. This physicochemical characteristic may limit its direct pharmacological applicability in vivo. However, these limitations are common among polyphenolic compounds and do not preclude their relevance as bioactive molecules. In this context, punicalin could be considered a lead structure for the development of optimized derivatives, formulation strategies, or delivery systems aimed at improving its pharmacokinetic properties while preserving its biological activity.

Future studies should aim to translate these in vitro findings into more complex preclinical models. Zebrafish xenograft models provide a unique platform for studying tumor–host interactions in vivo, allowing visualization of angiogenesis and metastatic dissemination. Implantation of breast cancer cells into zebrafish embryos can recapitulate early tumor growth, vascular remodeling, and cell invasion, offering a rapid and highly informative system for evaluating the anti-angiogenic and anti-metastatic potential of bioactive compounds such as punicalin [43,44].

In parallel, three-dimensional co-culture systems of tumor and endothelial cells could provide an intermediate step to dissect intercellular crosstalk under controlled conditions. These systems would enable detailed mechanistic studies of how punicalin modulates autophagy, ROS, and angiogenic signaling within the TME before proceeding to in vivo models.

Murine models remain essential to assess the impact of punicalin on tumor progression, angiogenesis, and metastasis in a mammalian system. For instance, the 4T1 model recapitulates triple-negative breast cancer characteristics, including rapid growth and spontaneous metastasis, making it suitable for evaluating both tumor cell-autonomous and microenvironment-mediated effects of therapeutic compounds [43].

It is expected that integrating in vitro co-culture assays, zebrafish xenografts, and murine models could allow a comprehensive preclinical evaluation of punicalin, providing insights into its efficacy and mechanisms of action on both tumor cells and the endothelial compartment. These approaches could also facilitate the identification of optimal dosing regimens, potential combinatorial strategies, and predictive biomarkers of response. This valuable information could contribute to open new therapeutic perspectives [45].

## 4. Materials and Methods

### 4.1. Cell Culture

The human TNBC cell line MDA-MB-231 was obtained from the American Type Culture Collection (ATCC, Rockville, MD, USA) and maintained in RPMI-1640 (Lonza, Basel, Switzerland).

The human microvascular endothelial cell line HMEC-1 was kindly provided by Dr. Arjan W. Griffioen (Maastricht University, Maastricht, The Netherlands) and cultured in MCDB-131 medium (Corning, Somerville, MA, USA) supplemented with 1 μg/mL hydrocortisone and 10 ng/mL endothelial growth factor-1 (EGF-1) (Sigma/Merck, Darmstadt, Germany).

The human umbilical vein endothelial cells (HUVECs) were purchased from Lonza (Basel, Switzerland) and cultured in EGM-2 medium, used up to a maximum passage number of 9.

All cultures were maintained at 37 °C under 5% CO_2_-enriched atmosphere.

Culture media were supplemented with 10% fetal bovine serum (FBS; Capricorn Scientific, Ebsdorfergrund, Germany), 1% penicillin/streptomycin, and 2 mM L-glutamine (BioWest, Kansas City, KS, USA). Cell culture plastics were supplied by Thermo Scientific Nunc (Thermo Fisher Scientific, Waltham, MA, USA).

Cells were routinely monitored for morphology and contamination and were used for experimentation at ~80% confluence. MDA-MB-231, HMEC-1 and HUVECs were routinely subcultured every three days at ratios of 1:6, 1:4 and 1:3, respectively.

### 4.2. Compounds and Treatment Preparation

Punicalin was purchased from Merck (Darmstadt, Germany) and reconstituted in dimethyl sulfoxide (DMSO) to generate stock solutions. Working concentrations were prepared by diluting stocks directly into complete culture medium for each cell line. Equivalent volumes of DMSO were added to control wells as vehicle controls in all assays. All treatments were carried out in complete media at the doses and incubation times specified in each experimental protocol.

### 4.3. Cell Viability Assay (MTT)

MDA-MB-231, HMEC-1, and HUVECs were seeded in 96-well plates at densities of 4 × 10^5^ cells/mL, 6 × 10^5^ cells/mL, and 4 × 10^3^ cells/mL, respectively, and incubated with serial dilutions (1:2) of punicalin, starting from a maximum concentration of 200 µM for 72 h. Cell viability was assessed using the MTT assay (5 mg/mL in PBS; Sigma/Merck). After a 4 h incubation, formazan crystals were dissolved with 0.04 N HCl in isopropanol, and absorbance was read at 550 nm using a microplate reader. IC_50_ values were calculated using GraphPad Prism (v8) and defined as the concentration of punicalin required to reduce cell viability by 50% compared to untreated control cells, serving as a quantitative indicator of the compound’s cytotoxic potency. Each concentration was tested in quadruplicate, and at least three independent experiments were performed (n = 3). Data are shown as mean ± standard deviation (SD).

### 4.4. Autophagy Detection by High-Content Screening

MDA-MB-231 and HMEC-1 cells were plated in 96-well plates at densities of 3.5 × 10^4^ cells/mL and 5 × 10^4^ cells/mL, respectively, and allowed to adhere for 24 h before treatment. Autophagic activity was examined under both basal conditions and nutrient deprivation using Earle’s Balanced Salt Solution (EBSS). Punicalin was applied at concentrations corresponding to 0.25×, 0.5×, 1×, and 2× IC_50_ values. Cells were stained with Hoechst 33342, CellMask Deep Red, and DAPGreen Autophagy Detection reagent (Thermo Fisher Scientific).

Live-cell imaging was carried out using the Operetta High-Content Imaging System (PerkinElmer, Waltham, MA, USA) over 4.5 h, acquiring images every 30 min and capturing four fields per well, under controlled temperature and CO_2_ conditions. Quantitative image analysis was performed with Harmony software (v4.8). Fluorescence intensities corresponding to cytoplasmic regions and autophagosomes were corrected for background using a defined pericellular area. Corrected autophagosomal fluorescence was calculated on a per-cell basis and averaged per condition, excluding mitotic cells and inaccurately segmented objects. Additional image post-processing was carried out using ImageJ software (v1.50).

A schematic overview of the experimental and analytical workflow is provided in Figure S1.

### 4.5. Oxidative Stress Assay by High-Content Screening

MDA-MB-231 and HMEC-1 cells were seeded in 96-well plates at densities of 3.5 × 10^4^ cells/mL and 5 × 10^4^ cells/mL, respectively. After 24 h, intracellular reactive oxygen species (ROS) levels were analyzed under basal conditions and following oxidative challenge. For induced oxidative stress, cells were exposed to 0.54 mM H_2_O_2_ for 15 min, after which the medium was replaced with fresh complete medium. Punicalin was tested at 0.25×, 0.5×, 1×, and 2× IC_50_ concentrations. Fluorescent labeling was performed using Hoechst 33342, CellMask Deep Red, and CellROX Deep Orange (Thermo Fisher Scientific).

Time-lapse acquisition was performed over 4.5 h, collecting images every 30 min and recording four fields per well using the Operetta system. Image quantification was conducted with Harmony software, measuring background-subtracted fluorescence intensity at the single-cell level. Mitotic cells and improperly segmented objects were excluded from analysis. Additional image post-processing was carried out using ImageJ software (v1.50).

A detailed representation of the experimental workflow is shown in Figure S2.

### 4.6. Fluorescent Probe Optimization

All fluorescent dyes were pre-optimized for working concentrations and cytotoxicity. Final assay conditions are described in Supplementary Table S1.

### 4.7. Cell Migration Evaluation by Wound Healing Assay

MDA-MB-231, HMEC-1 and HUVECs were seeded in 6-well plates and grown to full confluence. A scratch wound was created using a sterile 200 µL pipette tip, washed with PBS, and cells were incubated in fresh medium containing punicalin at 0.5×, 1× and 2 × IC_50_, or DMSO (vehicle control). Images were taken at 0, 4, 7, and 24 h using a Nikon Eclipse Ti microscope equipped with a DS-Ri2 camera (Nikon, Tokyo, Japan). The wound area was quantified using ImageJ software (v1.50), and migration was calculated as the percentage of wound closure relative to the initial area. Three independent experiments were performed (n = 3).

### 4.8. Tube Formation Assay

A 96-well plate was coated with 50 μL of Matrigel (10.5 mg/mL) (Corning, New York, NY, USA) at 4 °C and allowed to polymerize for 30 min at 37 °C. HMEC-1 cells were seeded at a density of 1.25 × 10^5^ cells/mL in MCDB-131 medium without FBS, whereas HUVECs were seeded at the same density in EBM-2 medium without FBS. Subsequently, 5 μL of different concentrations of the treatments were added to the experimental wells, and the plate was incubated for 7 h at 37 °C in a humidified atmosphere containing 5% CO_2_. After the incubation period, wells were examined and tube-like structures were evaluated. Images were captured using a Nikon DIAPHOT-TMD inverted phase-contrast microscope (Nikon, Tokyo, Japan).

The minimum inhibitory dose was defined as the lowest concentration of the compound that completely inhibited the formation of tubular structures compared to negative controls, which exhibited 100% tube formation. In each experiment, two replicates were performed for each concentration, and 20 µM toluquinol was used as a positive control [9]. At least three independent experiments were carried out (n = 3), and data were expressed as mean ± SD.

### 4.9. Statistical Analysis

Quantitative data are presented as mean ± standard deviation (SD) from at least three independent experiments. Prior to statistical analysis, data distribution was assessed using normality tests. Statistical comparisons were performed in GraphPad Prism software using either a two-tailed unpaired Student’s *t*-test or ANOVA, depending on the experimental design. Differences were considered statistically significant when *p* < 0.05. Significance levels are indicated as follows: * *p* < 0.05, ** *p* < 0.01, *** *p* < 0.001, and **** *p* < 0.0001.

Generative artificial intelligence (GenAI) tools were used to assist in the organization, structuring, and language refinement. Specifically, ChatGPT (OpenAI, San Francisco, CA, USA; GPT-5.2) was used. All scientific content, interpretations, data analysis, and final decisions regarding the manuscript were made by the authors, who critically reviewed and edited all AI-assisted content to ensure accuracy, originality, and consistency with the experimental results.

## 5. Conclusions

The results of the present study demonstrate that punicalin exerts selective, cell type-specific effects on tumor and endothelial cells across multiple molecular and functional endpoints within the tumor microenvironment. Punicalin induced cell type-dependent cytotoxic effects in MDA-MB-231 and HMEC-1 cells, while sparing normal endothelial HUVECs. Autophagy is strongly inhibited in HMEC-1 but remains unaffected in MDA-MB-231, whereas oxidative stress is reduced in MDA-MB-231 and modulated in a concentration- and condition-dependent manner in HMEC-1.

**Figure 8:**
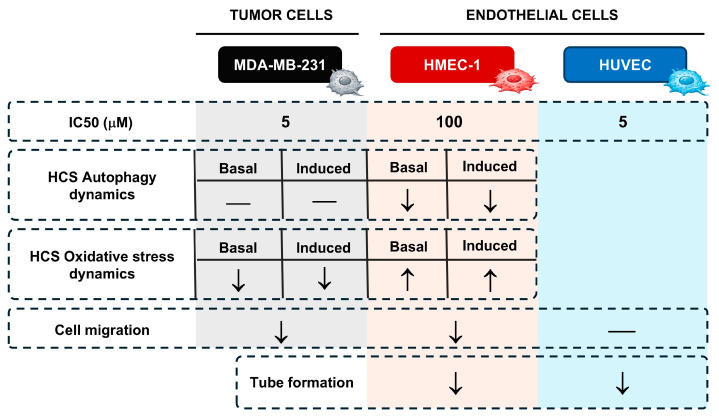
Summary of punicalin effects on MDA-MB-231, HMEC-1, and HUVECs. Schematic overview of the selective effects of punicalin on tumor and endothelial cells. Arrows indicate the direction and relative magnitude of the effect (↓ inhibition, ↑ increase).

Functional assays reveal partial inhibition of migration in MDA-MB-231 and HMEC-1 cells, and tube formation is significantly impaired in HMEC-1, with more modest effects in HUVECs. Collectively, these findings indicate that punicalin selectively targets tumor-associated endothelial cells while exerting limited effects on tumor cells and normal endothelium. A graphical summary is presented in Figure 8.

Altogether, our data highlight the relevance of targeting microenvironmental mechanisms—particularly autophagy and redox homeostasis in endothelial cells—to impair tumor angiogenesis. These results provide a strong rationale for further preclinical evaluation of punicalin as a natural compound with potential therapeutic value in breast cancer, especially within strategies aimed at disrupting tumor vascularization.

## Data Availability

The original contributions presented in this study are included in the article/Supplementary Materials. Further inquiries can be directed to the corresponding authors.

## Supplementary Materials

The supporting information (a pdf document containing the Supplementary Materials) can be downloaded at: https://www.mdpi.com/article/10.3390/ijms27031533/s1.

## Abbreviations

The following abbreviations are used in this manuscript:

ARPs	Autophagy-related proteins
ATCC	American Type Culture Collection
DMSO	Dimethyl sulfoxide
EBSS	Earle’s Balanced Salt Solution
EGF-1	Epidermal growth factor 1
ER	Estrogen receptor
FBS	Fetal bovine serum
GenAI	Generative artificial intelligence
HCS	High-content screening
HER2	Human epidermal growth factor receptor 2
HMEC-1	Human microvascular endothelial cells
HUVECs	Human umbilical vein endothelial cells
IC_50_	Half maximal inhibitory concentration
MDA-MB-231	Human triple-negative breast cancer cells
PBS	Phosphate-buffered saline
PDIA3	Protein disulfide isomerase A3
PR	Progesterone receptor
ROS	Reactive oxygen species
SD	Standard deviation
TME	Tumor microenvironment
TNBC	Triple-negative breast cancer
