# Supplementary material for: Punicalin Modulates Angiogenesis and Tumor Microenvironment-Related Processes in Triple-Negative Breast Cancer and Endothelial Cells
Source: Int J Mol Sci. 2026 Feb 4;27(3):1533. doi: 10.3390/ijms27031533 (PMC12898519; doi:10.3390/ijms27031533)
Supplement: Supplementary file 1 [file ijms-27-01533-s001.zip › ijms-4100393-supplementary.pdf]

Suplemmentary Materials

Punicalin Modulates Angiogenesis and Tumor Microenvironment–Related Processes in Triple-Negative Breast Cancer and Endothelial Cells

Maria Carmen Banqueri-Pegalajar[1,2], Joel D. Posligua-García[1,2], Carlos Ulises Cárdenas-Vela[1,2] Manuel Bernal[,2,3,*], Miguel Ángel Medina [1,2,3,*]

1 Department of Molecular Biology and Biochemistry, Faculty of Science, Universidad de Málaga, Andalucía Tech, E-29071 Málaga, Spain. mcbp@uma.es, joel.posgar@uma.es, ulisescardenas@uma.es, mbernal@uma.es, medina@uma.es

2 Molecular Bases of Biological Systems (SIBIUMA) Group, Instituto de Investigación Biomédica de Málaga y Plataforma en Nanomedicina-IBIMA Plataforma Bionand, Málaga, Spain.

3 Center for Biomedical Network Research in Rare Diseases (CIBERER), Instituto de Salud Carlos III, Madrid, Spain.

* Correspondence MBM, mbernal@uma.es; MAM, medina@uma.es

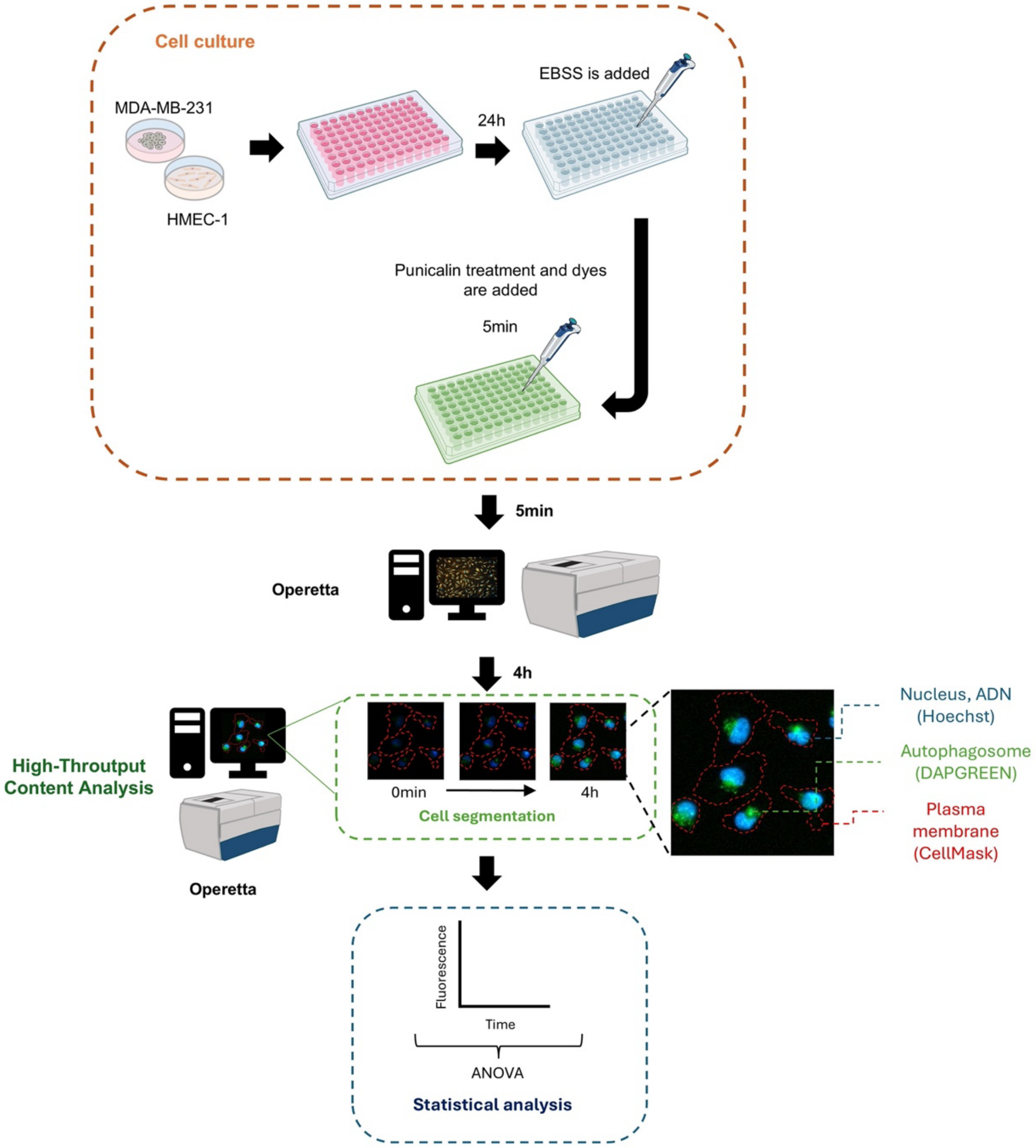

Figure S1. Workflow for the study of autophagy dynamics using High Content Screening. Schematic overview of the experimental setup used to monitor autophagy in MDA-MB-231 and HMEC-1 cells treated with punicalin under basal and starvation conditions. The diagram summarizes the treatment scheme, imaging schedule, and image analysis workflow performed with the Operetta High-Content Imaging System. Icons adapted from BioArt (National Institute of Allergy and Infectious Diseases) available at https://bioart.niaid.nih.gov/.

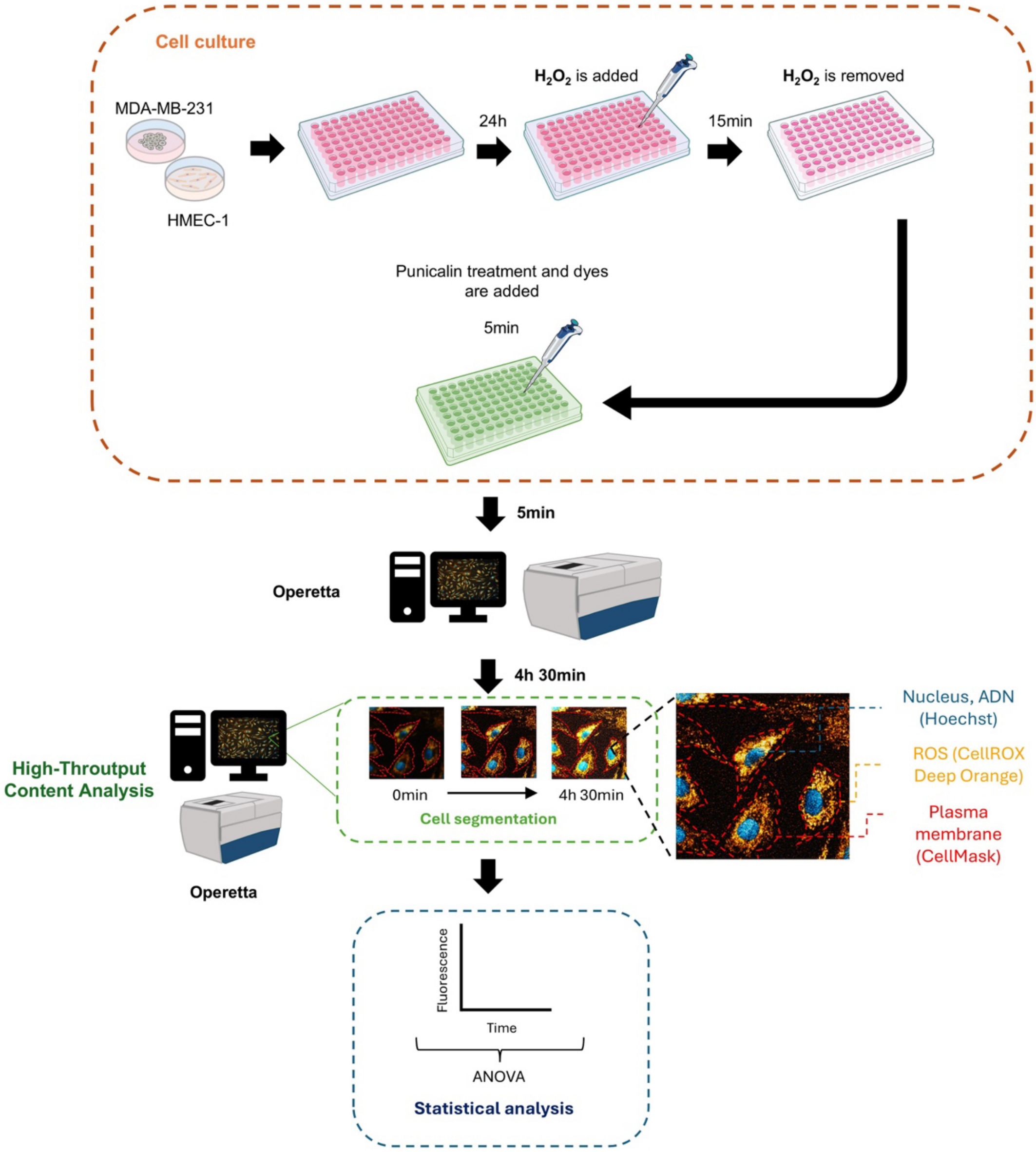

Figure S2. Workflow for the study of oxidative stress dynamics using High Content Screening. Schematic overview of the experimental setup used to monitor the oxidative stress in MDA-MB-231 and HMEC-1 cells treated with punicalin under basal and H_2O_2-induced conditions. The diagram summarizes the treatment scheme, imaging schedule, and image analysis workflow performed with the Operetta High-Content Imaging System. Icons adapted from BioArt (National Institute of Allergy and Infectious Diseases) available at https://bioart.niaid.nih.gov/.

Supplementary Table 1: Optimal conditions for the use of fluorescent probes

Dyes	Work concentration	Exposure time – Excitation height	Excitation and emission range	Channel	Target	Reference
**Hoechst 33342**	2.5 μm/ml	10ms – 1μm	Ex: 360 – 400 nm Em: 410 – 530 nm.	UV	DNA	H1399
**CellMaskTM Deep Red**	0.25 μm/ml	50ms – 1μm	Ex: 620-640nm – Em 650 – 760nm	Deep red	Plasma membrane	C10046
**DAPGreen Autophagy Detection**	0.1 μM	13ms – 1μm	Ex: 460 – 490 nm. Em: 500-550 nm.	Green	Autophagosome (LC3)	D67610
**CellRox Deep Orange**	50 μM	200ms – -3μm	Ex: 520 – 550 nm. Em: 560-630 nm.	Red	Reactive oxygen species (ROS)	C10443

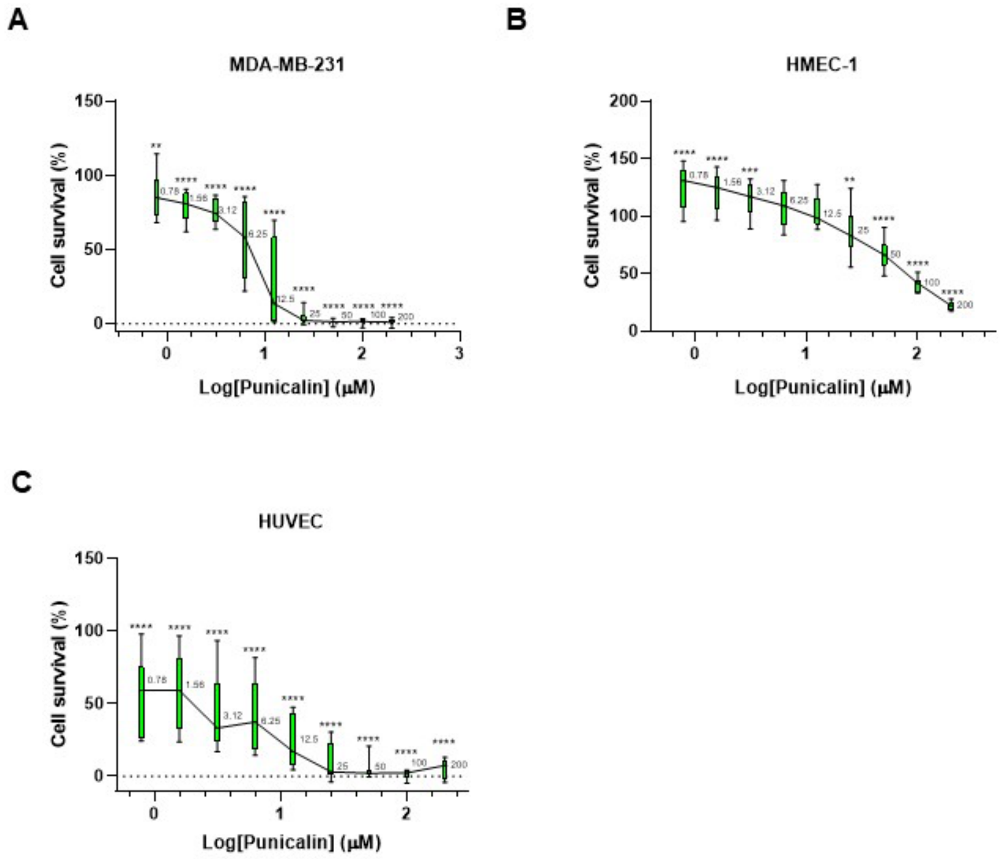

Figure S3. Dose–response analysis of cell viability with punicalin treatment. (A) MDA-MB-231 triple-negative breast cancer cells, (B) HMEC-1 microvascular endothelial cells, and (C) HUVEC primary endothelial cells were treated with increasing concentrations of punicalin for 72 h, and cell viability was assessed using the MTT assay. Data are shown as mean ± standard deviation (SD) from three independent biological experiments, each performed in quadruplicate.

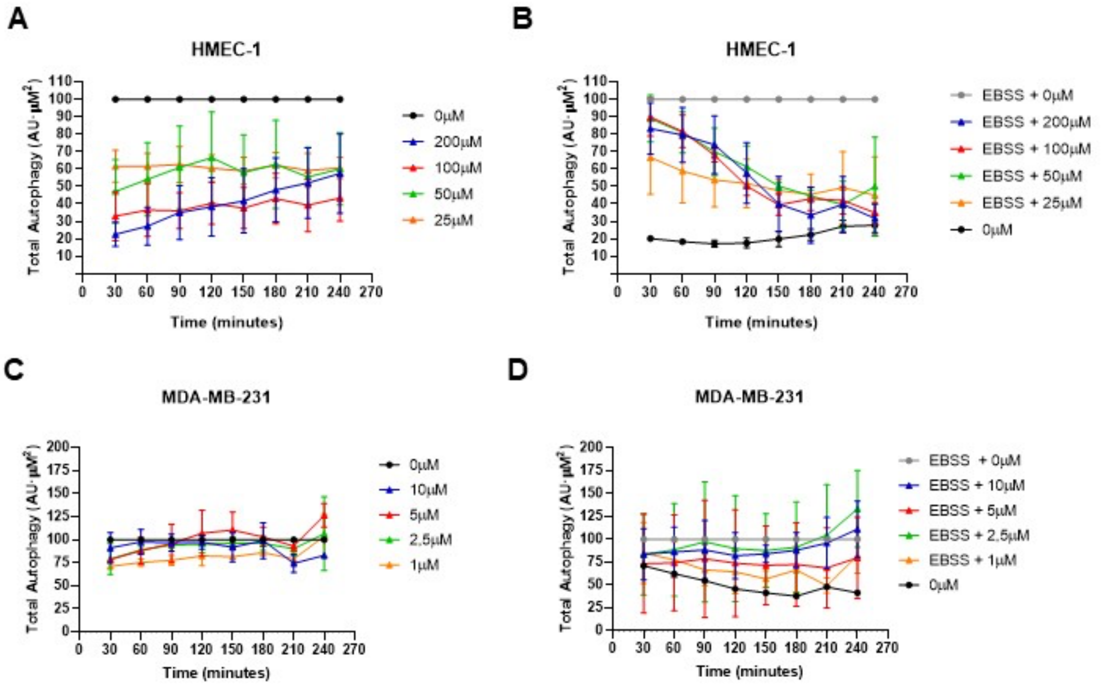

Figure S4. Quantitative analysis of autophagy dynamics in endothelial and tumor cells treated with punicalin. (A) Basal autophagic activity in HMEC-1 cells (B) EBSS-induced autophagy in HMEC-1 cells (C) Basal autophagic activity in MDA-MB-231 cells, (D) EBSS-induced autophagy in MDA-MB-231. Data represent mean ± SD from three independent experiments.

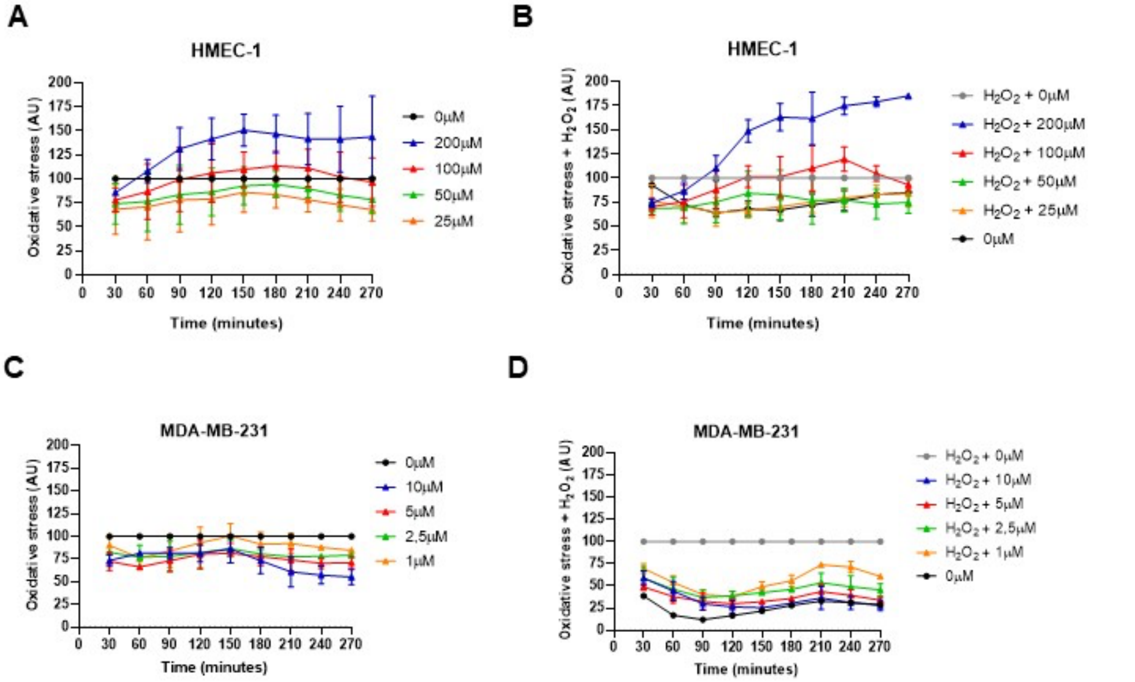

Figure S5. Quantitative analysis of oxidative stress dynamics in endothelial and tumor cells treated with punicalin. (A) Basal oxidative stress in HMEC-1 (B) H_2O_2-induced oxidative stress in HMEC-1 (C) Basal oxidative stress in MDA-MB-231 (D) H_2O_2-induced oxidative stress in MDA-MB-231.
